# Segmented Microfluidic Flow Reactors for Nanomaterial Synthesis

**DOI:** 10.3390/nano10071421

**Published:** 2020-07-21

**Authors:** Yujuan He, Ki-Joong Kim, Chih-hung Chang

**Affiliations:** 1 School of Chemical, Biological, and Environmental Engineering, Oregon State University, Corvallis, OR 97331, USA; heyuj@oregonstate.edu; 2 National Energy Technology Laboratory, U. S. Department of Energy, Pittsburgh, PA 15236, USA

**Keywords:** microfluidic reactors, segmented flow, nanomaterials synthesis, reaction kinetics, micromixer design

## Abstract

Microfluidic reactors have remarkably promoted the synthesis and investigation of advanced nanomaterials due to their continuous mode and accelerated heat/mass transfer. Notably, segmented microfluidic flow reactors (SMFRs) are an important class of microfluidic reactors that have been developed to accurately manipulate nanomaterial synthesis by further improvement of the residence time distributions and unique flow behaviors. This review provided a survey of the nanomaterial synthesis in SMFRs for the aspects of fluid dynamics, flow patterns, and mass transfer among and within distinct phases and provided examples of the synthesis of versatile nanomaterials via the use of different flow patterns.

## 1. Introduction

Microfluidic reactors have rapidly developed in the past few decades to achieve a sustainable and elaborate synthesis of advanced nanomaterials [1,2,3,4,5,6,7,8,9]. Academic research and industrial use of microfluidic reactors in different fields, such as chemistry, physics, and biology, have contributed to the creation and interdisciplinary applications of diverse nanomaterials. However, the application and scale-up production of microfluidic reactors have been constrained by their wall fouling and uneven axial residence time distribution (RTD) due to the parabolic velocity profiles of the microfluid. The uneven RTD usually limits the precise manipulation of reaction processes, such as crystal nucleation and growth, self-assembly, and intensification, that rely strongly on the uniform distribution of the temperature, pressure, and reactant concentration. The wall fouling that triggers microchannel blockages is detrimental for the maintenance of a stable and continuous process. Segmented microfluidic reactors (SMFRs) containing multiple phases have been introduced to address these issues. Compared with single-phase microfluidic reactors, SMFRs not only decrease the parabolic velocity profile and, therefore, significantly reduce the RTD and wall fouling but also accelerate mass and heat transfer due to their compartmentalization and unique flow behavior.

**Figure 1:**
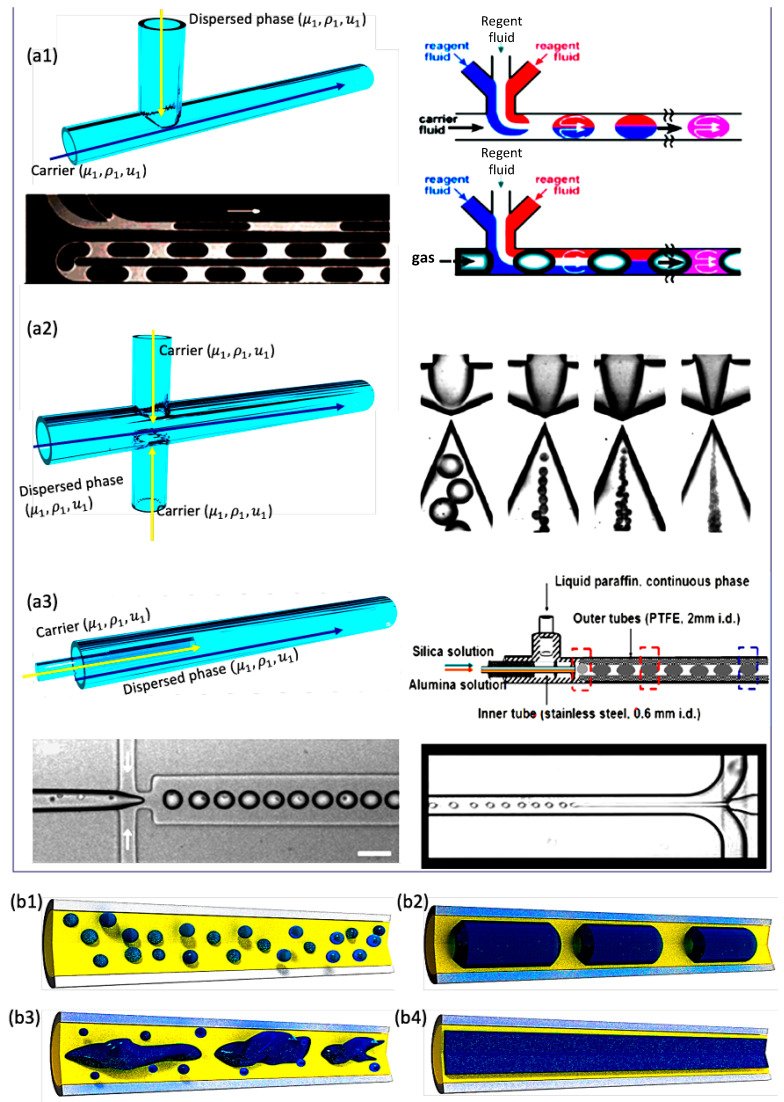
Schematic figures of and segmented-flow examples in (**a1**) T-junction (Reproduced from [19], with permission from American Chemical Society, 2005) (Reproduced from [20], with permission from Polymer, 2012), (**a2**) flow-focusing (Reproduced from [21], with permission from Royal Society of Chemistry, 2008), and (**a3**) injection configurations (Reproduced from [22], with permission from Elsevier, 2013). Schematic images of two-phases flow patterns—(**b1**) droplet/bubble flow, (**b2**) slug flow (Taylor flow), (**b3**) churn flow, and (**b4**) annular flow.

Segmented flow, also known as multiphase flow, has the advantages of small volume and large interfacial area for carrying out the chemical and biological synthesis, fluid analysis, and chemical separation [10,11,12,13,14,15,16,17,18]. This review focused on the synthesis of nanomaterials and understanding of the basic laws and advantages of SMFRs with respect to fluid dynamics, flow patterns, and mass transfer among and within distinct phases. The three main mixer configurations of forming segmented flow are the T-configuration, flow-focusing configuration, and injection configuration (Figure 1a–c). The compartmentalization in SMFRs is created by introducing immiscible fluids to form bubbles (gas in liquid), droplets (dispersed liquid in a carrier liquid), Taylor slugs, churn, and annular flow patterns (Figure 1a,b). SMFRs can be categorized mainly into gas–liquid SMFRs and liquid–liquid SMFRs based on their continuous and dispersed phases. The gas–liquid SMFRs are formed by gas and liquid, while two immiscible liquids, such as oil and water, comprise the liquid–liquid SMFRs. Both systems allow the reactions for the formation of nanomaterials either in the confined phase only or at the interfaces of the two phases. Examples of the implementation of versatile nanomaterials, such as metal, metal oxide, and chalcogenide materials, via different flow patterns have been discussed in the final section.

## 2. Fundamentals of Segmented Flow Reactor 

### 2.1. Fluid Dynamics

**Figure 2:**
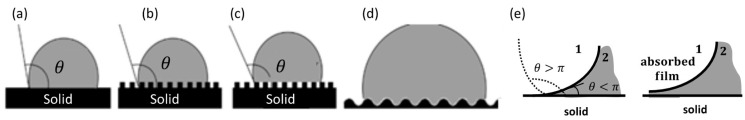
(**a**–**d**) Small droplets on surfaces with different morphologies (Reproduced from [25], with permission from Society of Wood Science and Technology, 2010). (**e**) Local configuration in the three-phase region. Left: the two fluid phases (1 and 2) and the solid wall intersect at the contact line with angle θ; right: the fluid phases are separated by an adsorbed film (Reproduced from [26], with permission from Royal Society of Chemistry, 2006).

In segmented flow, the forces around or in each discrete compartment define the compartment shape, stability, dispersion, and interaction with the surrounding environment. These forces in SMFRs mainly are the interfacial force (surface tension), viscous force, inertia force, and gravity and depend on the properties and velocities of the fluids and the geometries and dimensions of the microfluidic devices. Typically, the static fluid phases under low Reynolds number are equilibrium compartments, where the pressures within each phase are uniform but differ from each other to sustain a curved interface defined by the Young–Laplace equation Δp_1-1/g-1_ = σκ (σ is the interfacial tension, and κ is the local mean curvature). Three interfacial tensions σ_12_, σ_2s_, σ_1s_ between the immiscible phases 1 and 2 and between the fluids and microchannel fundamentally balance the compartments in the microfluidic system and are related according to σ_12_ cosθ = σ_1s_ − σ_2s_, where θ is the contact angle between the fluid and the microchannel surface that is defined by the liquid wettability of the surface and can be varied using surfactants or different surface morphologies (Figure 2). The relationship between wettability and surface behavior has been thoroughly described in the literature [23,24,25]. 

Dimensionless numbers used in fluid mechanics are beneficial for investigating the dominant forces in segmented flow. These numbers describe the interactions between different forces and classify the dominant forces of distinct segmented-flow-behavior. Table 1 summarizes the commonly used dimensionless numbers. The critical values of these dimensionless numbers are essential for defining the transition between two different configurations of segmented flows because the interplay of the forces controls the formation of compartments in microfluidics. Taking the critical capillary number Ca_c_ ≈ 10^−2^ of gas–liquid systems as an example, it can be seen that the shear force dominates segmented formation when Ca > Ca_c_, while the shear stress is negligible when Ca < Ca_c_, resulting in a squeezing regime. A recent study [27] has demonstrated that an empirical correlation based on the dimensionless capillary number and liquid slug length can accurately describe the recirculation inside the liquid slugs, and the slug length is affected by both the capillary number and the flow rate ratio in a gas–liquid system. The following section has discussed two essential aspects of SMFRs, namely, the flow pattern and mass transfer. The mixing in the Taylor slug, which is an important SMFRs for the synthesis of nanomaterials, has been discussed in the following section.

### 2.2. Flow Patterns

#### 2.2.1. Gas–Liquid SMFRs

**Figure 3:**
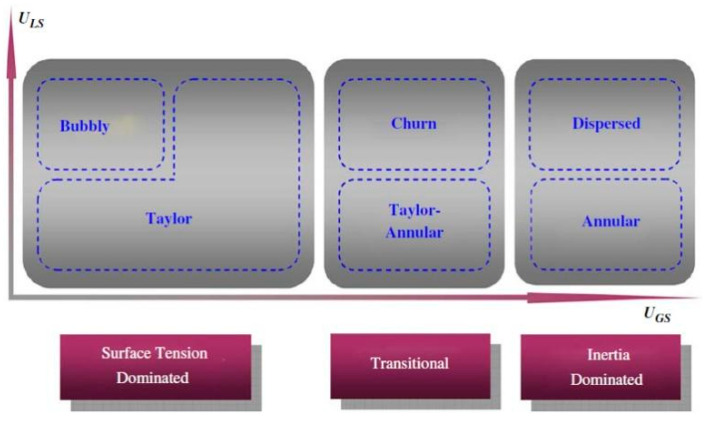
The general classification of flow patterns (Reproduced from [32], with permission Elsevier, 2009).

Various flow patterns have been designed to create diverse reaction conditions for nanomaterial synthesis in the gas–liquid segmented flow [28,29,30,31]. Two-dimensional flow-pattern-maps demonstrate that the segmented flow pattern transforms with the variation of the hydrodynamic parameters, such as fluid velocities [32], Weber numbers [33], and Capillary numbers [34]. The general tendency of flow patterns has been extensively studied, even though the creation of a universal flow-pattern-map is not feasible due to the intrinsic geometrical differences among the different fluid systems and various wall effects due to difference in the wettability, contamination, and roughness of the channel surface. Shao et al. proposed a general classification of flow patterns based on the dominant forces (Figure 3) [32]. At high liquid velocity, the gas flow transforms from bubbles to Taylor flow (slug flow) and then to churn and dispersed flow with increasing gas velocity. At low liquid velocity, the gas flow transitions from Taylor flow to Taylor-Annular and then to annular flow with increasing gas velocity. Wang et al. [34] proposed a transition prediction model in which the capillary number is a function of the Reynolds numbers of the gas (Re_G_) and liquid (Re_L_) phases (Equation (1)).

(1) Ca=a(θ180°)b(ReGReL)c  

In Equation (1), θ is the contact angle between the fluid phases with the channel wall, a, b, and c are the constants of the transition boundary of the flow region, which, for example, are 4 × 10^−4^, 1, −0.76, respectively, for the transition boundary between the slug and slug-annular flow [34].

**Figure 4:**
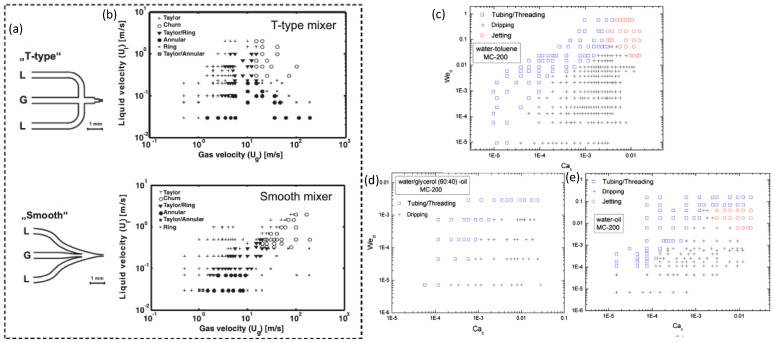
(**a**) Two types of inlets of segmented microfluidic reactors (SMFRs). (**b**) Flow-patterns of the N_2_/H_2_O system for the “T-type” and “Smooth” inlets (Reproduced from [38], with permission from Wiley, 2006). Flow pattern maps at/near the inlet junction based on Ca_c_ and We_d_ for (**c**) water-toluene, (**d**) water-oil, and (**e**) water/glycerol-oil two-phase flows in a rectangular channel of 200 μm × 200 μm (Reproduced from [40], with permission Elsevier, 2017).

Previous studies have discussed segmented-flow behavior in terms of several parameters, including fluid velocity, wall wettability, channel size, and inlet geometry. Shao et al. [32] suggested that small microchannel size could shift all flow patterns in the plot of U_LS_ vs. U_GS_ (Figure 3) to the left due to the increasing dominance of surface tension over the inertia forces in smaller channels that restrained the coalescence disruption from the bubble surface. This perspective is consistent with the report of Saisorn et al. [35], who investigated the flow pattern, void fraction, and pressure drop of the air-water flow for different channel diameters. Saisorn et al. also found that the annular and dispersed regions were barely affected by the channel dimensions, but the waves appearing around the gas core became thin with decreasing channel diameter. The study by Yue et al. [36] considered the effect of the channel size on the absorption of CO_2_ into water. Choi et al. [37] proposed a C value in the Lockhart and Martinelli’s correlation. The C value decreased with the reduced hydraulic diameter of a rectangular microchannel, reflecting a weakened interaction between the two phases. In addition to the geometrical dimension of the microchannels, the wettability of the channel surface also plays an essential role in segmented-flow behavior because the interface of two phases, which is defined by the Young–Laplace equation, depends strongly on the contact angle between the fluid and the channel wall. A decrease in the wettability of the channel wall can inhibit the liquid spreading on the tube wall and lead to the preference to form droplets, rivulet, and multi rivulet flows instead of annular flow. Additionally, the inlet geometry is also critical for the careful control of the flow patterns. According to Haverkamp et al. [38], the different angles of the inlet channel resulted in significant divergence in the flow pattern. For example, the “smooth” junction formed both Taylor and annular flows at a liquid velocity of approximately 0.05 m/s and gas velocity of approximately 1 m/s (Figure 4b); by contrast, the “T-type” junction only created annular flow in the same velocity region (Figure 4a). The data variation of annular and churn in the “T-type” junction was higher than that in the “smooth” junction. Saito et al. also reported that junctions with higher angles initiated stable droplet generation more quickly than the junctions with lower angles [39].

#### 2.2.2. Liquid–Liquid SMFRs

Liquid–liquid flow has a similar flow-pattern map to the gas–liquid flow and has been investigated based on the fluid velocities and the dimensionless numbers Re, We, Ca, and Bo [41,42,43]. Most flow patterns of liquid–liquid SMFRs are discussed based on the Weber number because its flow regime mainly depends on the domination of the interfacial force and the inertial force. Wu et al. reported the flow pattern of the commonly used liquid–liquid systems, namely, the water-toluene, water-oil, and water/glycerol-oil systems, in rectangular channels [40]. Figure 4c–e presents the typical flow patterns in a rectangular channel with a dimension of 200 μm × 200 μm, and the flow patterns in terms of different channel dimensions, fluidic velocities, and liquid ratios can be found in the article by Wu et al. [40]. Zhao et al. [44] discussed the flow pattern transition of kerosene-water within the T-junction of a rectangular microchannel and depicted the interfacial-tension dominated, transitional- and -inertia dominated flow regions at both the T-junction and the microchannel. The flow region as a function of the capillary lengths was studied by Jovanovic et al. to show the flow-pattern transformations between the disparate locations, such as the transition from parallel flow at the beginning to annular flow [45]. Numerical modeling has been employed to simulate the flow patterns of the SMFRs of multiple organic phases as a function of channel size, fluids properties, and slug length [41]. The meandering channel, as an important microchannel structure, has also been applied in liquid–liquid SMFRs. Sarkar et al. [46] discussed the effects of the serpentine channel on the flow patterns using a water-succinic acid-butanol system. The high-frequency deformation from repeated bends of the serpentine microchannel introduced interfacial instabilities to each compartment and, therefore, decreased the diffusion length. Although several articles have reported on the studies of the liquid–liquid segmented flow [44,45,47,48,49,50], a comprehensive understanding of its flow pattern and the interactions between two immiscible liquids is still lacking. 

### 2.3. Mass Transfer

Due to uniquely confined compartments and flow patterns, SMFRs can provide distinct mass transfer for the synthesis of nanomaterials. SMFRs dramatically enhance the mass transfer within and among the compartments due to the inner flow recirculation and the small volume but a large interfacial area of each compartment. In each compartment, vigorous mixing aroused by the inner flow recirculation can dispel the parabolic velocity profile and diminish the uneven mass distribution on axial. The isolated mass transfer within the compartments contributes to protecting the microchannel from the direct contact with reaction precursors or nanomaterials and efficiently avoids fouling and blockage [51,52,53]. The following sections have described several commonly used mass-transfer-models of segmented flow. 

#### 2.3.1. Interfacial Mass Transfer

**Figure 5:**
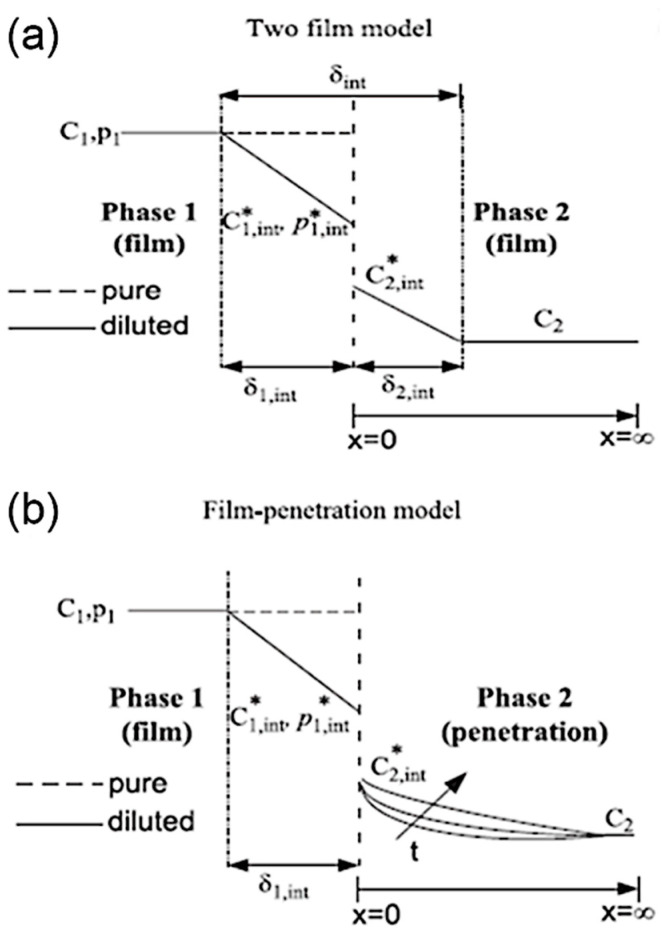
Schemes of the concentration profile at the gas–liquid and liquid–liquid interfaces. (**a**) Two-film model and (**b**) film-penetration model (Reproduced from [54], with permission from Elsevier, 2011).

Numerous models have been established to describe and predict the interfacial mass transfer (IMT) in SMFRs over the last few decades. Two basic types of IMT models, namely, the stagnant film model and the film penetration model (Figure 5), were first introduced by Whitman in 1923 and by Higbie in 1935, respectively [54]. The stagnant film model considers the interface as a stagnant film with a thickness σ_i_, where a concentration gradient drives the molecular diffusion. The film penetration model assumes the element of the surface of the first phase is fully exposed to the second phase at a finite time prior to the second phase, reaching a constant concentration in the bulk of the first phase. The two IMT models and the fluid flow models are usually combined to provide suitable descriptions of different SMFR systems. The mass transfer coefficient k_L_a is critical for characterizing the mass transfer performance and can be expressed based on the solute concentration as given below.

$k_{L}a=\frac{1}{\tau }\ln\left(\frac{C_{2}^{*}-C_{2}^{\mathrm{in}}}{C_{2}^{*}-C_{2}^{\mathrm{out}}}\right)$, when the solute concentration is high (e.g., C_1_ is constant)

$k_{L}a=\frac{1}{\tau \left(\frac{1}{{K\varepsilon }_{1}}+\frac{1}{1-\varepsilon_{1}}\right)}\frac{C_{2}^{\mathrm{in}}-C_{2}^{\mathrm{out}}}{\Delta C_{\mathrm{lm}}}$, when the solute concentration C_1_ is low, and the volume fraction is not negligible. $\varepsilon_{1}=\frac{Q_{1}}{Q_{1}+Q_{2}}$, *Q*_i_ is the volumetric flow rate of the i^th^ phase. C_i_* is the equilibrium concentration of phase i. ΔC_lm_ is the logarithmic mean concentration difference [54]. 

Although the stagnant film and film penetration models are useful for understanding the mass transfer between the interfaces, they are simplified models with the assumptions of stagnant film or fully exposed interfacial areas, which are ideal conditions and can hardly describe the surface refreshing due to fluidic recirculation in each SMFRs compartment. With the development of current technologies, the IMT can be experimentally determined because in situ characterizations of interfacial mass transfer is now feasible. The study of the absorption and reaction of the CO_2_ gas and liquid is a common approach for investigations of the IMT in SMFRs because the CO_2_ dissolved in a liquid can give rise to pH variation but can barely vary the liquid volume. Yue et al. [28] utilized two-film models to determine the mass transfer coefficient (k_L_a) of absorbing CO_2_ into a buffer solution of NaHCO_3_/Na_2_CO_3_/NaOH in a rectangular microchannel with a hydraulic diameter of 667 µm. They found that the mass transfer rate of CO_2_ into the buffer solution was much higher than the reaction rate. Therefore, the detection of the CO_2_ mass transfer was unaffected by the chemical reaction, and the k_L_a at the various velocities of the gas and liquid flows was obtained. Nieves-Remacha et al. [55] determined the k_L_a by a modified penetration model using bubble slip velocity instead of the bubble velocity in a stagnant liquid. They indicated that the overall mass transfer coefficient k_L_a increased with power consumption and gas holdup. The model-derived k_L_a values matched well the experimental values at low liquid velocities but deviated from the experimental values at a high liquid velocity. This disparity might be due to the instability in the fluid at a high liquid velocity that was not sufficiently considered in the mathematical model. The CO_2_ absorption in NaOH in a meadow microtube and an arc-shaped channel was studied by Tan et al. [30]. Tan et al. studied the mass transfer as a function of the gas-slug-length and the flow pattern around the curvatures by detecting the pH at multiple locations in a microchannel. They demonstrated that the k_L_a increased significantly with decreasing curvature radius, and the difference between the inner and outer channels of the curve-shaped channel could give rise to more vigorous circulation within the liquid compartment. Yao et al. pointed out that raising the system pressure to 3 MPa could decrease the bubble length but increased both the gas absorption and mass transfer coefficient of the CO_2_-water system [56]. By visualizing the CO_2_-bubble-volume shrinkage with a time resolution of milliseconds, Saito et al. obtained instantaneous mass transfer coefficients and found that the velocity of the bubble hemisphere was accelerated due to the zigzag motion that contributed to the high renewal rate of the liquid on the bubble interface [57]. The numerical model simulated using COMSOL has been applied to evaluate the CO_2_ absorption profile in NaOH [58]. The model includes the reaction between NaOH and CO_2_ that is usually ignored in most studies because its rate is much higher than the rate of mass transfer. The COMSOL model has demonstrated that the CO_2_ absorption rates increase significantly when the reaction is considered since the CO_2_ consumption contributes to a consistently large driving force across the mass transfer interface.

The studies on the IMT of liquid–liquid SMFRs have been carried out in the last two decades. Dessimoz et al. [59] used the reaction between trichloroacetic acid and NaOH, in which the NaOH only could dissolve in the water phase, but trichloroacetic acid could diffuse from the organic phase to the water phase. They determined the k_L_a of liquid–liquid slug and parallel flows in the range of 0.2–0.5 s^−1^ using the two-film model. In the study by Jovanovic et al. [45], the mass transfer between an organic mixture (butanol and toluene) and water in different flow patterns were investigated by a constant volume semi-batch model and experiments at various residence times. They demonstrated that the surface-to-volume ratio reached more than 150,000 m^2^/m^3^ by the bubbly flow that could contribute to a prominent k_La_. Fricke et al. revealed an additional activation energy barrier for the adsorption and desorption of triethylamine at the interface of n-decane and water and finally obtained accurate adsorption rate constants by interpreting the interfacial-tension-measured data using a mathematical model [60].

#### 2.3.2. Mixing in the Taylor Slugs

The distinctive mixing and mass transfer within each compartment of Taylor slugs has attracted much attention in the field of reactions and crystallization of nanomaterials. The Taylor vortices, which are the recirculation inside the confined Taylor slug, can diminish the broad residence time distribution (RTD) and give rise to turbulence inside each compartment and, therefore, significantly increase the mixing efficiency. The annular and parallel flow only has a few or no internal circulation [45], while the recirculation inside the Taylor slugs is obvious and can be visualized by particle image velocimetry [61]. In the straight horizontal channel, the circulatory motion is symmetrical with respect to the channel center, and the internal recirculation is closely related to the fluid properties [62] as well as the slug length [58]. As studied by both numerical and experimental methods [58,62,63,64], the fluid velocities can influence the axial dispersion and the recirculation inside the segmented flow. At low velocities, the interfacial force is not strong enough to induce full recirculation, and conversely, the faster convection within the full recirculation at high velocity usually contributes to better mixing performance. Muradoglu et al. established a computational model to study the influences of the Peclet number, the capillary number, and segment size on the axial dispersion in the gas–liquid segmented flow using a finite-volume-and-front-tracking method [65]. They defined the diffusion-controlled, convection-dominated, and diffusion-convection regimes based on the Peclet number. Muradoglu et al. stated that the capillary number played an essential role in the axial dispersion in the diffusion-controlled regime but had minor effects on the convection-dominated regime. For the chemical reaction, Termühlen et al. indicated that the homogeneity of slug length was crucial because it influenced the recirculation in each compartment [66]. They found that both synthetic gas supply via channel pressure and T-junction mixer were favorable for the homogeneous slug length and stable compartments. The computational fluid dynamics (CFD) model of Yang et al. has demonstrated that the diffusion coefficient increases with decreasing slug length due to the diminished slug volume and increased specific interfacial area [67].

**Figure 6:**
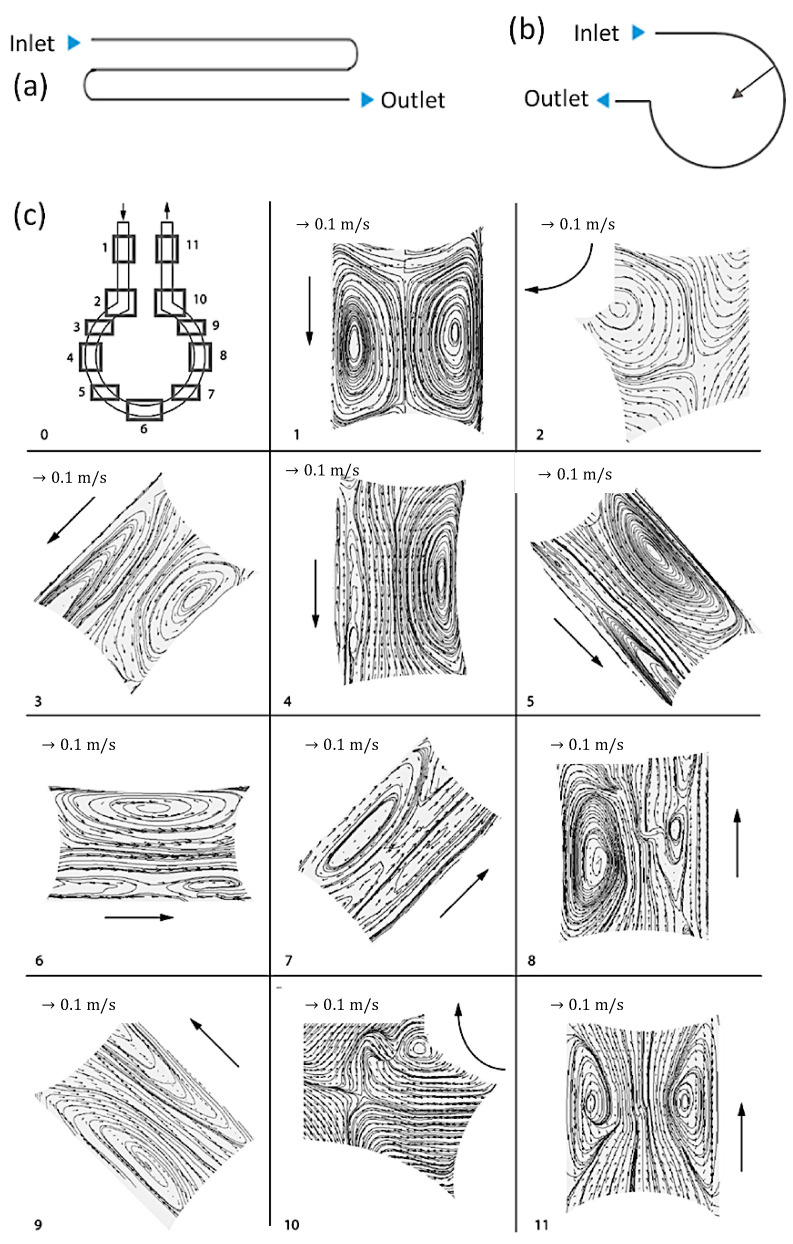
(**a**) Common meadow channel and (**b**) arc-shaped channel. (**c**) Various flow patterns with different channel curvatures (Reproduced from [64], with permission from Elsevier, 2009).

The mixing in Taylor slugs can be further enhanced by engineering the channel structure and introducing external disturbance, such as ultrasonication. Meandering channel (Figure 6a) is a crucial path structure for enhanced mixing because the axisymmetric circulation flow in each compartment breaks and substances redistribute when the flow is deformed around the channel curve and secondary flow (Dean vortices) forms in different planes (Figure 6c). According to the study by Tan et al. [30], the increased radius of the arc-shaped channel (Figure 6b) dramatically increased the k_L_a value of both interfacial and inner mass transfer of the slugs. Typically, longer slugs show better mass transfer than shorter slugs in the meandering channel as the turning angle of shorter slugs is much smaller than that of the longer slugs [64]. Kurt et al. combined the helically coiled structure and 90° bends to achieve rapid internal diffusion and up to 14% higher conversion compared to straight tubes [68]. Samuel et al. provided optimized riblet structures of a fast-swimming shark on the channel surface to reduce the drag of segmented flow by lifting the vortices formed in the turbulent flow that generated transverse shear stresses [69]. Introducing external disturbance is another efficient approach to increase the mixing in Taylor slugs. Dong et al. reported an increment of the overall mass transfer coefficient by 3.3–5.7 times using high ultrasound power density that provided stable and uniformly distributed intensification mechanism—fierce surface wave oscillation of the slug bubble [70].

## 3. Nanomaterials Synthesis by Segmented Flow

SMFRs have been widely applied and have become essential for obtaining uniform and high-quality functional materials due to their distinctive fluidic mechanics’ characteristics [71,72,73]. SMFRs successfully prevent blocking issues experienced by single-phase reactors of nanomaterials [74]. Nanomaterial morphologies and properties have been tuned by varying the SMFRs parameters, such as velocity and compartment length. This section highlights the recent work of two typical reaction routes in SMFRs—reactions within compartments and at the interface.

### 3.1. Reaction within Compartments of Taylor and Bubbly Flow

**Figure 7:**
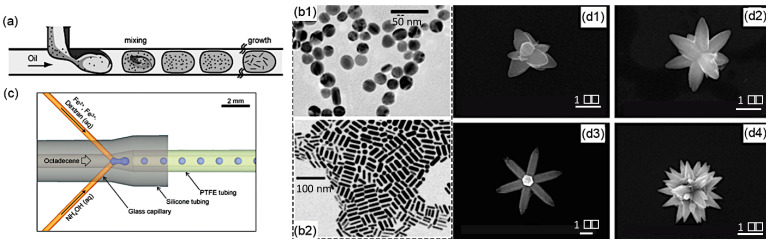
(**a**) Scheme of droplet-based synthesis in a microreactor. TEM images of (**b1**) Au nanospheres and (**b2**) Au nanorods (Reproduced from [76], with permission Wiley, 2009). (**c**) SMFRs with injector joint to create reactant droplets (Reproduced from [79], with permission from Royal Society of Chemistry, 2012). (**d1**–**d4**) ZnO nanoparticles (NPs) in diverse shapes (Reproduced from [81], with permission from Elsevier, 2004).

Compartments in Taylor and bubbly/droplet flows are naturally well-defined microenvironments and are widely employed for nanomaterial synthesis. As discussed above, the recirculation in compartments promotes convective mass transfer and reduces axial dispersion and RTD, which can provide accurate control of the nanomaterial size, size distribution, and morphology. The spatial confinement avoids the cross-contamination induced by physical contact between the reagents and the channels and, therefore, ensures the reproducibility and scalable production of microfluidic reactors. The reactions performed in the dispersed or continuous phases have been used to create diverse nanomaterials with distinct properties. The synthesis of metal nanomaterials, such as gold (Au), has benefited from the advantages of the reactions within compartments. Cabeza et al. discussed the influence of channel wettability and reaction temperature on the synthesis of gold nanoparticles (NPs) [75]. Duraiswamy et al. reported on the tunable growth of anisotropic metal nanocrystals in 2009 (Figure 7a) [76]. They obtained gold nanoparticles in the shape of spheres/spheroids and rods by varying the reagent concentration and the flow rate ratio of oil to aqueous reagent streams (Figure 7b). The obtained nanoparticles presented different surface plasmonic and optical resonances in the visible–near-infrared (NIR) spectral range. Both liquid–liquid and gas (air)–liquid methods for Ag NPs have been studied by Kumar et al. [77] in a unidirectionally expanding spiral SMFRs. They observed that the size distributions of NPs in both systems were highly dependent on the slip velocity and slug size that dominated the nature of internal mixing in the reactant phase, and gas–liquid SMFRs tended to generate smaller particle size than liquid–liquid SMFRs. In addition to metal nanomaterials, large amounts of metal oxide and chalcogenide nanomaterials were synthesized within the compartments. Li et al. created zinc oxide (ZnO) in different flower-like shapes from the homogeneous reactions provided by a water-oil Taylor flow reactor (Figure 7d) [78]. Kumar et al. [79] applied the injector joint to create precursor droplets in a flow of octadecene (Figure 7c) and produced dextran-coated Fe_2_O_3_ NPs. Yen et al. first utilized the excellent mixing and narrow RTD of gas–liquid SMFRs to eliminate the regional heat and mass gradient of the batch injection processes and synthesized monodispersed cadmium selenide (CdSe) quantum dots with no clogging [80].

Compared to single-phase microfluidic reactors, the shear force induced by segment movements and internal recirculation is another unique property of compartments for creating various nanomaterial morphologies. As shown in previous sections, both numerical models and particle image velocimetry have depicted highly localized high-shear regions in compartments. The groups of Moffitt and Sinton reported that the shear-induced particle break-up acting as a competitor of the collision-induced coalescence played an essential role in the control of the self-assembly of quantum dot-block copolymer colloids [82,83]. The colloid size was tuned from 40 nm to 140 nm by varying the shear rate through changing the gas-to-liquid flow ratio and flow rate. Xu et al. directly compared the flow control of the multiscale structure in a single-phase staggered herringbone and Taylor-flow reactor to demonstrate that the formation of the crystalline core of the PCL-b-PEO nanocomposites was mainly influenced by the high shear rather than by rapid mixing [84]. Due to the high shear at high flow rates, the copolymer chains self-assembled into spherical nanocomposites instead of into lamellae that are generated at low shear. The specific morphologies, such as spool generated from the shear control, have been employed to manipulate the loading and releasing speeds of drugs and dyes by the group of Matthew G. Moffitt [85,86]. 

A careful choice of the continuous and dispersed phase is necessary for the synthesis of nanomaterials because the shear force and mixing within compartments rely strongly on the channel wettability and rheological properties of each phase. Cabeza et al. studied the effects of different dispersed phases, such as air, toluene, and silicon oil, on the size and size distribution of gold nanoparticles [75]. At identical flow rates, air as the dispersed phase led to the strongest recirculation in the aqueous reagent phase and contributed to the smallest size variation that indicated the narrowest RTD. They pointed out that an increase in the viscosity of the dispersed phase resulted in a reduction in the slip velocity among the phases and the channel that reduced the magnitude of recirculation. Although the gas/liquid at low viscosity provides strong circulation, such phases usually display high evaporation pressure and become unstable at elevated temperatures, which is detrimental for many nanomaterial reactions. Marre et al. [82] proposed a high-pressure process to maintain the low-viscosity solvent as the liquid phase at the high reaction temperature. However, high-pressure routes can enhance the mass transfer at the interfaces among the phases that can diminish the boundaries between the compartments, broaden the RTD, and attenuate the control of the nanomaterial size and morphology. An appropriate phase choice for the reaction is essential for the nanomaterial synthesis within the compartments.

### 3.2. Interfacial Reaction

Interfacial reactions among the different phases and/or distinct streams have attracted increasing attention for the synthesis of novel artificial nanomaterials with advanced functionalities. Placing reactants separately in different phases and/or streams allows the reactions to occur mainly at the interfaces and be primarily driven by diffusion. The reactions at the interfaces of SMFRs can be categorized into three main groups based on the flow type and are the Taylor-flow, annular/parallel-flow, and bubbly/droplet-flow reactions. Many intermediate, one-dimensional (1D), and two-dimensional (2D) nanomaterials that cannot be achieved by conventional reactors have been generated by manipulating the diffusion at the interfaces, by altering the flow rate and rate ratio of the phases and/or streams of SMFRs [87,88]. The tunable diffusion-limited condition is not only useful in rationalizing new artificial and transient nanomaterials but also valuable for revealing the mechanism and evolution of the various reactions, such as redox, epitaxial crystal growth, and self-assembly processes.

In addition to enhancing mixing, the recirculation in Taylor-flow also contributes to the frequent refreshing of the interfaces between two adjacent compartments and renews the concentration gradient at interfaces to promote diffusion and reaction efficiency. Varying the recirculation magnitude can regulate the diffusion for different synthesis purposes. Meaningfully, the compartmentalized confinement allows the reaction with toxic or strongly oxidizing reagents to be carried out at safe and mild conditions. Hydrogen (H_2_) and carbon monoxide (CO) have been used as “clean” reductants to obtain metal nanomaterials, such as 3–25 nm gold nanoparticles [89] and 3–5 nm silver nanoparticles [90]. Switching the gas phase from oxygen to CO has enabled the modification of the palladium nanostructure growth from 1D (nanorods) to 2D (nanosheets) [87]. Liquid–liquid interfaces have also been utilized to form gold nanoparticles by Kulkarni et al. [91]. They showed that the particle size and size distribution were modified by changing the channel wettability from hydrophilic to hydrophobic, which converted the continuous and dispersed phases. They also reported on the particle evolution from the branch-like shape to hexagonal bipyramidal shape using a hydrophilic channel.

**Figure 8:**
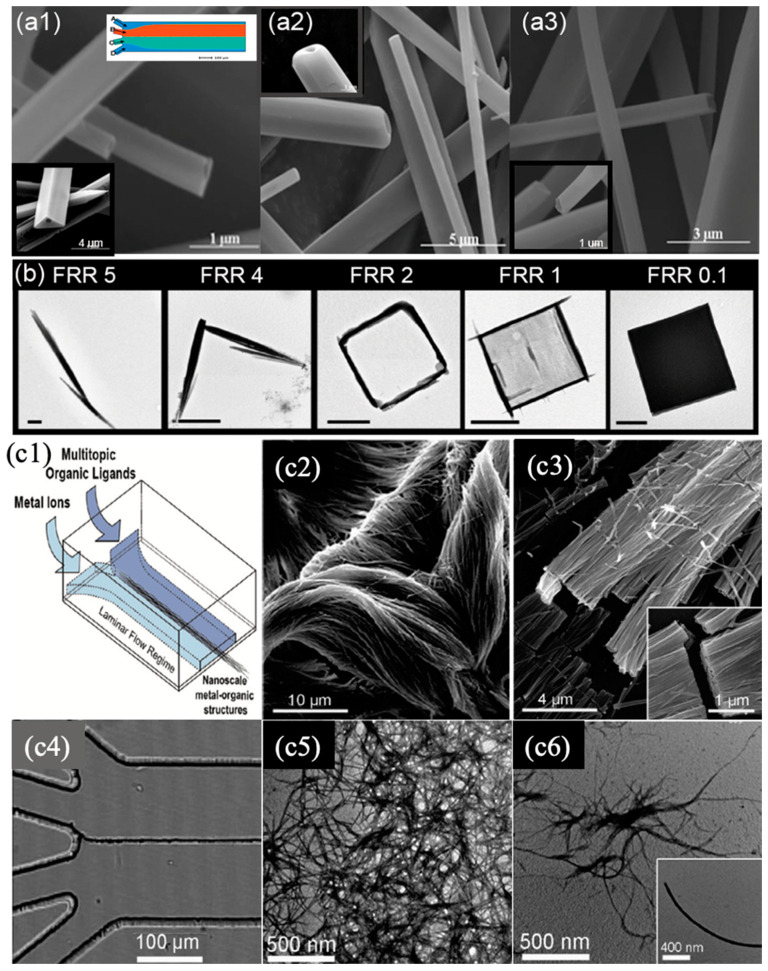
(**a**) SEM images of hollow hybrid wires prepared at the flow rate ratios of 1 (**a1**,**a2**) and 2 (**a3**). (The insets in a1, a2, and a3 are SEM images at high magnification. The top right inset in a1 is a schematic illustration of the microfluidic device of four parallel streams.) (Reproduced from [92], with permission from Wiley, 2010). (**b**) The sequence of TEM images of crystals of the coordination polymer fabricated in the microfluidic device at different flow rate ratios (FRR), showing trapped crystalline phases that ranged from needles to hollow frames to plate-like crystals (left to right). Scale bars: 1 μm (Reproduced from [88], with permission from Wiley, 2016). (**c1**) Schematic image of fabrication of 1D coordination polymer nanostructures using laminar flow in a microfluidic platform. Cu(II)-Asp nanofibers fabricated in the microreactor. (**c2**,**c3**) SEM images of nanofiber bundles synthesized at different concentrations of precursors: (**c2**) 150 mM and (**c3**) 15 mM Cu(NO_3_)_2_∙3H_2_O. (**c4**) Optical microscopy image, showing the guided assembly of 1D nanostructure bundles created at the interface between aqueous Ag(I) metal ions and Cys solutions. (**c5**) TEM images of the resulting bundles of Ag(I)-Cys (**c5**) and Zn(II)-$4^{\prime }$40-bipy (**c6**) nanofibers just after their elution from the chip. The inset shows a high-magnification image of a single Zn(II)-$4^{\prime }$40-bipy nanofiber. (Reproduced from [93], with permission from American Chemical Society, 2011).

Annular/parallel flow with two or multiple laminar streams is a dynamic processing technology that displays an enlarged interfacial area and provides precise spatial and temporal control over the concentration profiles and mass transport. Similar to Taylor-flow SMFRs, the diffusion-limited condition in annular/parallel facilitates the formation of exceptionally structured nanomaterials, particularly nanofibers and nanotubes with the lengths of micrometers that currently cannot be obtained in other SMFRs. Puigmartí-Luis et al. employed a four-streams parallel flow to create tubular and hollow wires by varying the ratio of the flow rates of the carrier and reactant streams [92]. In their design (Figure 8a), the two acetonitrile streams acted as carrier streams to deliver the two reactant streams and as-prepared wires smoothly in the microchannel. The synthesized hybrid wires with triangular, square, and round profiles exhibited the typical electrical response in the dielectrophoretic characterizations. In another article by Puigmartí-Luis et al., a similar flow platform was applied to form one-dimensional coordination polymer nanostructures (Figure 8c1–c6) [93]. The works of Puigmartí-Luis et al. demonstrated that the size and morphology of wires and tubes were tuned by altering the ratio of the flow rates and reactant concentration; however, barely any variation was obtained by using different flow rates with the same rate ratio. Interestingly, Hiroaki et al. used one of the streams in an annular flow as the template to fabricate meter-long core-shell hydrogel microfibers, encapsulating extracellular matrix (ECM) proteins and differentiated cells or somatic stem cells [94]. In this work, the shell stream transformed into a gel and finally encapsulated the biological substances along the microchannel. The fibers were assembled into the higher-order cellular assembly for transplant into diabetes mellitus. Rubio-Martinez et al. used different diffusion timescales controlled by flow rate ratio to capture the transient crystals-needles, square frame, and square sheet in the crystal growth process of a coordination polymer (Figure 8b) [88]. In addition to nanowires, nanorods, and nanofibers, the annular flow has also been applied for obtaining spherical nanoparticles, such as TiO_2_ [95]. The outer stream of annular flow is an excellent barrier between the reagent stream and the channel wall using which the Fe_2_O_3_ nanoparticles with a diameter of 5 ± 0.5 nm have been protected from oxidation and have displayed extraordinary magnetic properties [96].

Reactions at the interfaces of the droplets offer opportunities for using the droplets as templates to form versatile materials. The reagent absorption on the surface of the droplets is usually followed by a reaction of forming a solid shell [97]. Substances, such as biocatalysts, dyes, and drugs, can be loaded into the core-shell structures by carrying these substances in droplets [98,99,100]. These routes have created few nanomaterials because the current techniques can hardly generate stable nanodroplets and prevent their coalescence. 

### 3.3. Others

**Figure 9:**
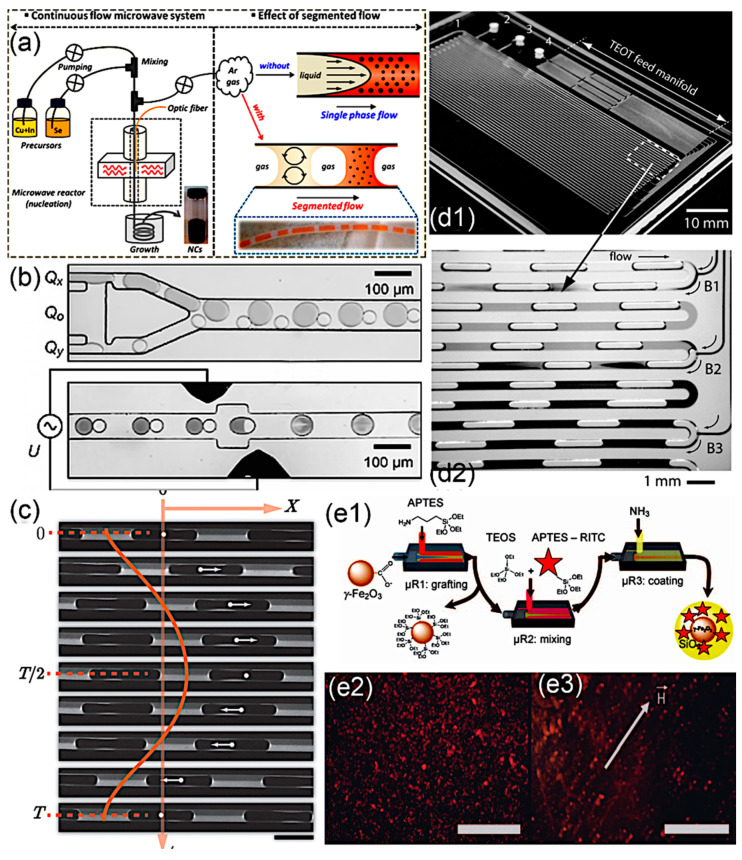
(**a**) Schematic illustration of the SMFRs coupled with microwave irradiation for the synthesis of chalcogenide nanomaterials (Reproduced from [102], with permission from ACS Publication, 2014). (**b**) Electrocoalescence-assisted SMFRs for droplet merging (Reproduced from [105], with permission from Wiley, 2008). (**c**) Experimentally obtained fluorescence images of one complete oscillation cycle, scale bar is 1 mm (Reproduced from [103], with permission from Royal Society of Chemistry, 2014). (**d1**) Photograph of the microfluid with multistep addition of TiO_2_ shells on colloidal SiO_2_ (Reproduced from [109], with permission from Wiley, 2007). (**e1**) Scheme for the continuous synthesis of fluorescent core/shell MNP (magnetic nanoparticles)/silica nanoparticles; fluorescence micrographs of silica-coated iron oxide nanoparticles (**e2**) in the absence of an external magnetic field and (**e3**) in the presence of an external magnetic field, showing the formation of chain-like structures. Scale bars: 50 μm (Reproduced from [110], with permission from Wiley, 2009).

With the increasing technological development, SMFRs have been further exploited and improved for more rigorous and sophisticated nanomaterial synthesis. External features, such as microwave and ultraviolet (UV) irradiation, electrical voltage, and oscillation, have been introduced to either facilitate precise energy input or manipulate the mixing in SMFRs. SMFRs with inner recirculation are applicable solutions of the sparkling issue in single-phase microfluidic reactors coupled with the microwave because this issue is caused by the in-situ deposition of the nanomaterials on the inner channel wall at the microwave zone. Chalcogenide binary PbSe NPs [101] and ternary CuInSe_2_ NPs [102] have been synthesized using microwave-assisted SMFRs. Chang’s group employed microwave-assisted SMFRs to develop the route for the separation of crystal nucleation and growth stages and achieved precise control over the reaction by fine-tuning of the parameters and stage separation (Figure 9a) [102]. Applying oscillation to the multiphase flow requires subtle control of the movement and a careful choice of the involved phases. Abolhasani et al. used the periodical manipulation of the pressure at the device inlet and outlet via square wave signals generated by nonwetted solenoid valves to create a consistent back-and-forth motion in the segmented flow (Figure 9c) [103]. Different mixing profiles were achieved by varying the oscillation amplitudes and oscillation frequencies, and thereby various morphologies of gold nanobars were created. An indirect ultrasonication zone, established by Jiang et al., successfully varies the crystal seed generation rate during the operation independent of mass flow rate [104]. Instead of maintaining compartments’ isolations, Frenz et al. [105] coalesced the droplets to form droplet pairs by applying an electrical field between two on-chip electrodes (Figure 9b). The coalesced droplet pairs precipitated Fe_2_O_3_ NPs in a rapid manner (millisecond) and ensured the reproducibility of the reaction. Recently, Du et al. applied passive picoinjection via a venturi junction to accomplish the drop fusion and, therefore, the accurate addition of reagent for the synthesis of BaSO_4_, CaCO_3_ vaterite, and gold nanoparticles [106]. This passive picoinjetion efficiently avoided the fouling in the inlet junction and realized the controllable crystal precipitation of CaCO_3_ vaterite and gold nanoparticles. Additionally, external light sources, such as UV illumination coupled with a photoinitiator, have been used as prominent energy-inputs of green chemistry in the microfluidic system [107]. In the segmented flow, external light sources have allowed nanomaterials to be synthesized at mild temperatures, avoiding the complexities of fluids phase transfer. Toit et al. reported that variation in the UV exposure time and intensity at a given condition resulted in a nonmonotonic effect on the particle size and increased the particle size of the gold nanoparticles, respectively [108].

Core-shell nanomaterials are an important class of nanomaterials, which have benefited from the advantages of SMFRs. Compared to conventional single-material NPs, core-shell NPs possess enhanced and stable properties that are useful in electrical, photonic, and chemical fields [111]. One crucial class of core-shell nanomaterials utilize a chemically and physically passive material as the shell to protect the functional materials. Silica (SiO_2_), zinc sulfide (ZnS), and yttria (Y_2_O_3_) with high thermal, chemical, and mechanical resistance are commonly applied as the protecting shell of metal, metal oxide, and chalcogenide nanomaterials. The SiO_2_ shell contributes to avoiding any unspecific aggregation of NPs and provides a hydrophilic surface for further reaction and stable dispersion in the water phase [110]. Abou-Hassan et al. produced the γ-Fe_2_O_3_@SiO_2_ NPs with a size of 40 nm from a gas–liquid annular flow that effectively applied the surrounding gas to prevent the wall deposition of γ-Fe_2_O_3_ and scaled-down the hydrolysis and condensation dimensions by focusing the precursor flow. In addition to serving as a passive layer for protecting inner materials, shells, such as poly(DL-lactide-co-glycolide) (PLGA) [112], are used to alter the surface chemistry. The as-prepared CdSe/ZnS colloids cannot be dispersed into nonpolar organic solvents because of the tri-n-octylphosphine oxide surface ligand. For a stable dispersion of CdSe/ZnS colloids in polar solvents, self-assembling PLGA droplets with the size in the range of 100–200 μm (Figure 9e1) generated by a microfluidic junction are used to encapsulate thousands of CdSe/ZnS quantum dots and form a miscible surface for the polar solvent. Similarly, forming vesicles by surfactants PLGA in a microchannel, Valencia’s group [100] produced a lipid shell around both drug and QD (quantum dot), obtaining a homogeneous NPs coating. By controlling the mixing velocity and PBS concentration, the weight ratio between lipid and PLGA could be varied around the inner core. Another surface-modification example of the core-shell structure is the work by Gong et al. [99], who synthesized a shell containing Fe_2_O_3_ NPs out of an aspirin solution. Additionally, the core-shell structure is also beneficial for enhancing the specific properties of materials [109,112,113]. For example, the fluorescent intensity of CdSe NPs was dramatically enhanced by the ZnS shell (Figure 9e2) [112]. Khan et al. integrated multistep reagent additions into the gas–liquid segmented flow (Figure 9d) to obtain functional TiO_2_ with SiO_2_ template for a uniform size distribution [109].

## 4. Conclusions

In conclusion, SMFRs have greatly advanced the synthesis of nanomaterials due to their unique flow patterns and motion that avert broad RTD and wall fouling. This review discussed the fluid dynamics, flow patterns, mass transfer, and applications of the synthesis of functional nanomaterials in various SMFRs systems. Compared to single-phase microfluidic reactors, SMFRs provide a confined reaction environment for precise control of the morphology, size, purity, and, therefore, functions of materials. The nanomaterials obtained using SMFRs possess outstanding electrical, magnetic, and optical capabilities. However, many untapped aspects of SMFRs still need to be explored. The multiple phases of SMFRs usually give rise to more instabilities in the system and require judicious fluid control and device development. The vaporization and increased diffusion between immiscible fluids at elevated temperatures or low pressure still prevent the use of SMFRs in reactions for the synthesis of nanomaterials.

## Figures and Tables

**Table 1:** Important dimensionless numbers in segmented microfluidic reactors (SMFRs).

	Name	Definition and Numerical Equation	Comment
1	Reynold Number (Re)	Re=Inertial forceViscous force=ρUdDhμ	Re = We/Ca, ρ: kinetic viscosity, U_d_: mean velocity of the object relative to the fluid, D_h_: hydraulic diameter, μ: density of the relative fluid
2	Capillary Number (Ca)	$\mathrm{Ca}=\frac{\mathrm{Viscous}\;\mathrm{force}}{\mathrm{Interfacial}\;\mathrm{force}}=\frac{{\mu U}_{d}}{\sigma }$	σ: interfacial force
3	Bond Number (Bo)	$\mathrm{Bo}=\frac{\mathrm{Gravitational}\;\mathrm{force}}{\mathrm{Interfacial}\;\mathrm{force}}=\frac{\Delta {\rho \mathrm{gD}}_{h}^{2}}{\sigma }$	∆ρ: density difference between the two immiscible fluids, g: gravity
4	Weber Number (We)	$\mathrm{We}=\frac{\mathrm{Inertial}\;\mathrm{force}}{\mathrm{Interfacial}\;\mathrm{force}}=\frac{{\rho U}_{d}^{2}D_{h}}{\sigma }$	-
5	Sample Fraction (r_dc_)	$r_{\mathrm{dc}}\;=\;\frac{{\dot{Q}}_{d}}{{\dot{Q}}_{d}+{\dot{Q}}_{c}}$	${\dot{Q}}_{d}$: flow rates of the dispersed fluid, ${\dot{Q}}_{c}$: flow rates of the carrier fluid
6	Peclet Number (Pe)	$\mathrm{Pe}=\frac{\mathrm{Advective}\;\mathrm{transport}\;\mathrm{rate}}{\mathrm{Diffusibe}\;\mathrm{transport}\;\mathrm{rate}}$ $=\frac{U_{d}D_{h}}{D},=\frac{U_{d}D_{h}}{\alpha }$	D: mass diffusion coefficient
7	Fourier Number (Fo)	$\mathrm{Fo}=\frac{\mathrm{Diffusive}\;\mathrm{mixing}\;\mathrm{time}}{\mathrm{Residence}\;\mathrm{time}}$ $=\frac{\mathrm{Dt}}{D_{h}^{2}},=\frac{\alpha t}{D_{h}^{2}}$	Dt: diffuse mixing time, α: thermal diffusion defined as α = k/(ρc_p_), k: thermal conductivity, c_p_: heat capacity
